# Individualized Therapeutic Environments for Pain Management in Children with Autism Spectrum Disorder: A Scoping Review

**DOI:** 10.3390/children13080979

**Published:** 2026-07-23

**Authors:** María Fernández-Guarido, María Pilar Diéguez-Poncela, Laura Ruiz-Azcona

**Affiliations:** 1 Internal Medicine Section, Hospital Sierrallana, B. Ganzo s/n, 39300 Torrelavega, Spain; 2 Pediatric Emergency Section, Hospital Universitario Marqués de Valdecilla, Avda. Valdecilla s/n, 39008 Santander, Spain; 3 Departamento de Enfermería, Universidad de Cantabria, Avda. Valdecilla s/n, 39008 Santander, Spain; 4 Global Health Research Group, Universidad de Cantabria, Avda. Valdecilla s/n, 39008 Santander, Spain; 5 Grupo Investigación en Enfermería, IDIVAL-Instituto de Investigación Sanitaria Valdecilla, Avda. Cardenal Herrera Oria s/n, 39011 Santander, Spain

**Keywords:** autism spectrum disorder, pain management, pain measurement, pediatric nursing, child

## Abstract

**Highlights:**

**What are the main findings?**

- Evidence indicates that pain perception and expression in children with autism spectrum disorder are heterogeneous and frequently differ from those observed in neurotypical peers, complicating pain recognition.
- The literature consistently supports individualized, multidimensional pain assessment that integrates behavioral observation, caregiver reports, and validated assessment instruments.

**What are the implications of the main findings?**

- A personalized approach to pain assessment may improve the recognition and management of pain in children with autism spectrum disorder across healthcare settings.
- Future research should prioritize the validation of autism-specific pain assessment instruments and the evaluation of interventions that support evidence-based pain management.

**Abstract:**

Background/Objectives: Children with autism spectrum disorder (ASD) present unique challenges in pain assessment and management because of differences in communication, sensory processing, and pain expression, increasing the risk of pain underrecognition and inadequate treatment. This scoping review aimed to map and synthesize current evidence on pain processing, expression, assessment, and management in children with ASD, identify available pain assessment tools and interventions, and examine the contribution of individualized therapeutic environments to pain management. Methods: This scoping review was conducted according to the Preferred Reporting Items for Systematic Reviews and Meta-Analyses Extension for Scoping Reviews (PRISMA-ScR). PubMed, Scopus, and Web of Science were systematically searched for studies published between January 2020 and December 2025. Search strategies combined Medical Subject Headings (MeSH) and free-text terms. Two reviewers independently screened studies extracted data using a standardized form, and synthesized findings narratively. No formal methodological quality appraisal was undertaken, consistent with PRISMA-ScR recommendations. Results: Included studies demonstrated substantial heterogeneity in pain perception and expression, with atypical behavioral responses, sensory differences, and communication difficulties frequently hindering pain recognition. Individualized, multidimensional pain assessment integrating behavioral observation, caregiver reports, and validated assessment tools was consistently supported. Sensory adaptations, tailored communication strategies, caregiver involvement, distraction techniques, and virtual reality showed potential to improve pain-related experiences and reduce procedural distress. However, evidence remained predominantly observational, methodologically heterogeneous, and limited by few psychometrically validated ASD-specific assessment instruments and the absence of standardized clinical protocols. Conclusions: Current evidence supports individualized, multidisciplinary pain assessment and management for children with ASD. Nevertheless, substantial evidence gaps remain, highlighting the need for validated ASD-specific assessment tools, standardized clinical protocols, and high-quality studies evaluating pharmacological, non-pharmacological, and technology-assisted interventions.

## 1. Introduction

Autism Spectrum Disorder (ASD) is a neurodevelopmental condition characterized by persistent impairments in social communication and interaction, as well as restricted and repetitive patterns of behavior, interests, or activities. Its clinical presentation is highly heterogeneous, ranging from individuals with significant intellectual and language impairments to those with average or above-average cognitive functioning. This variability reflects the underlying complexity of the disorder and contributes to a broad spectrum of healthcare needs across the lifespan [1].

In recent decades, increasing attention has been paid to improving the understanding of ASD clinical manifestations, associated comorbidities, and implications for healthcare delivery, particularly in pediatric populations, where early identification and intervention are critical for optimal developmental outcomes. Research has also highlighted a growing global prevalence of ASD and the need for more tailored healthcare approaches [2].

In parallel, pediatric pain research has advanced through the development of multiple theoretical models of pain perception and processing. However, the integration of both fields remains limited, and evidence specifically addressing pain in children with ASD is still scarce [3].

In this population, typical neurodevelopmental processes interact with ASD-related alterations. Evidence suggests that neurobiological differences in ASD affect sensory regulation, leading to atypical stimulus processing that directly influences pain perception [4]. Communication and social interaction difficulties, core features of ASD, further complicate pain expression. Nevertheless, the literature remains inconsistent regarding pain processing in children with ASD. While some studies report sensory hypersensitivity and increased pain-related distress, others suggest reduced behavioral responses to painful stimuli or difficulties in pain communication rather than altered nociception itself. This heterogeneity reflects differences in study populations, outcome measures, and assessment methods, highlighting the complexity of interpreting pain experiences in this population [5,6,7].

Lack of awareness of these characteristics may negatively affect care quality, contributing to adverse patient experiences, increased resistance to care, underestimation of pain, and delays in treatment initiation [8]. Although several validated pediatric pain scales exist, no specific tools are available for children with ASD, limiting accurate assessment and intervention selection [9,10].

Because pain experiences in children with ASD are influenced not only by biological mechanisms but also by sensory, communicative, behavioral, and environmental factors, there is increasing interest in individualized therapeutic environments that integrate environmental adaptations, personalized communication, caregiver participation, and behavioral support. Nevertheless, the concept remains inconsistently defined in the literature, and the effectiveness of these approaches has not been comprehensively synthesized. [11]. Several non-pharmacological interventions adapted to children with ASD, including sensory adaptations, distraction techniques, and behavioral strategies, have shown promising results in improving pain-related experiences. However, the available evidence remains heterogeneous and is predominantly based on observational studies, limiting definitive conclusions regarding their effectiveness [12]. Family involvement, particularly by parents and caregivers, is considered an important component of individualized pain management, as caregivers can facilitate communication, provide behavioral insight, and support emotional regulation. However, evidence regarding its independent clinical impact remains limited [13]. Common strategies include deep breathing, transcutaneous electrical nerve stimulation, and distraction techniques, with growing evidence supporting virtual reality interventions [14,15,16].

Multidisciplinary, patient-centered care is essential. Nursing professionals play a key role in pain assessment, reassessment, and implementation of non-pharmacological strategies. Although previous reviews have examined individual aspects of pain assessment or non-pharmacological interventions in children with ASD, the available evidence remains fragmented and does not provide an integrated overview of pain assessment tools, pharmacological and non-pharmacological management strategies, and the contribution of individualized therapeutic environments within a pediatric context. Consequently, a comprehensive synthesis of the available evidence is lacking, leaving important uncertainties regarding current clinical practice and future research priorities [17,18].

Given these evidence gaps, a scoping review is warranted to systematically map the available literature, identify knowledge gaps, and provide a comprehensive synthesis to inform clinical practice, policy development, and future research in pediatric pain management for children with ASD. Accordingly, this scoping review aimed to map and synthesize the existing evidence on pain assessment and management in children with ASD, identify available pain assessment tools and pharmacological and non-pharmacological interventions, and explore the role of individualized therapeutic environments in supporting pain management and coping within this population.

## 2. Methods

This scoping review was conducted and reported in accordance with the Preferred Reporting Items for Systematic Reviews and Meta-Analyses Extension for Scoping Reviews (PRISMA-ScR) guidelines [19].

### 2.1. Search Strategy

A comprehensive literature search was performed in the following electronic databases: PubMed, Scopus, and Web of Science (WoS). The search covered studies published between January 2020 and December 2025, a period selected to capture the most recent evidence on advances in pain assessment, individualized therapeutic approaches, and emerging technologies for pain management in children with ASD. This timeframe was chosen to ensure that the review reflected contemporary clinical practice and recent developments in autism-informed pediatric care. Search filters were limited to publication date and language (English or Spanish), whereas no restrictions were applied regarding study design.

The search strategy combined Medical Subject Headings (MeSH) and free-text terms related to autism spectrum disorder, pain assessment and management, pediatric populations, nursing, caregivers, and healthcare. The search included the following terms: “Autism Spectrum Disorder”, “Pain Management”, “Pain Perception”, “Pediatrics”, “Child”, “Nursing Research”, “Caregiver”, “Pediatric Nursing”, and “Health Care”. Boolean operators (“AND” and “OR”) were adapted to the syntax of each database. The complete database-specific search strategies are provided in Supplementary Material S1.

### 2.2. Eligibility Criteria

Studies were included if they:

- Focused on pediatric populations diagnosed with ASD.
- Addressed pain perception, pain expression, pain assessment, or pain management.
- Examined pharmacological and/or non-pharmacological interventions, including therapeutic environments, sensory adaptations, or nursing care approaches.

Studies were excluded if they:

- Focused exclusively on pharmacological treatment without relevance to pain assessment, nursing care, or broader therapeutic context.
- Were opinion articles, editorials, conference abstracts, or non-peer-reviewed publications.
- Did not report pain-related outcomes in pediatric populations.

Given the broad objective of this scoping review, studies with different methodological designs were considered eligible. The anticipated methodological and clinical heterogeneity was addressed through narrative thematic synthesis rather than quantitative synthesis.

### 2.3. Study Selection and Data Extraction

All retrieved records were imported into a reference management software, and duplicate records were removed prior to screening. Study selection was conducted independently by two reviewers in two sequential stages. First, titles and abstracts were screened according to the predefined eligibility criteria. Subsequently, the full texts of potentially eligible studies were independently assessed for inclusion. Disagreements were resolved through discussion until consensus was reached. To minimize selection bias, both reviewers independently applied the same predefined eligibility criteria throughout the screening process, ensuring consistency in study selection.

**Figure 1:**
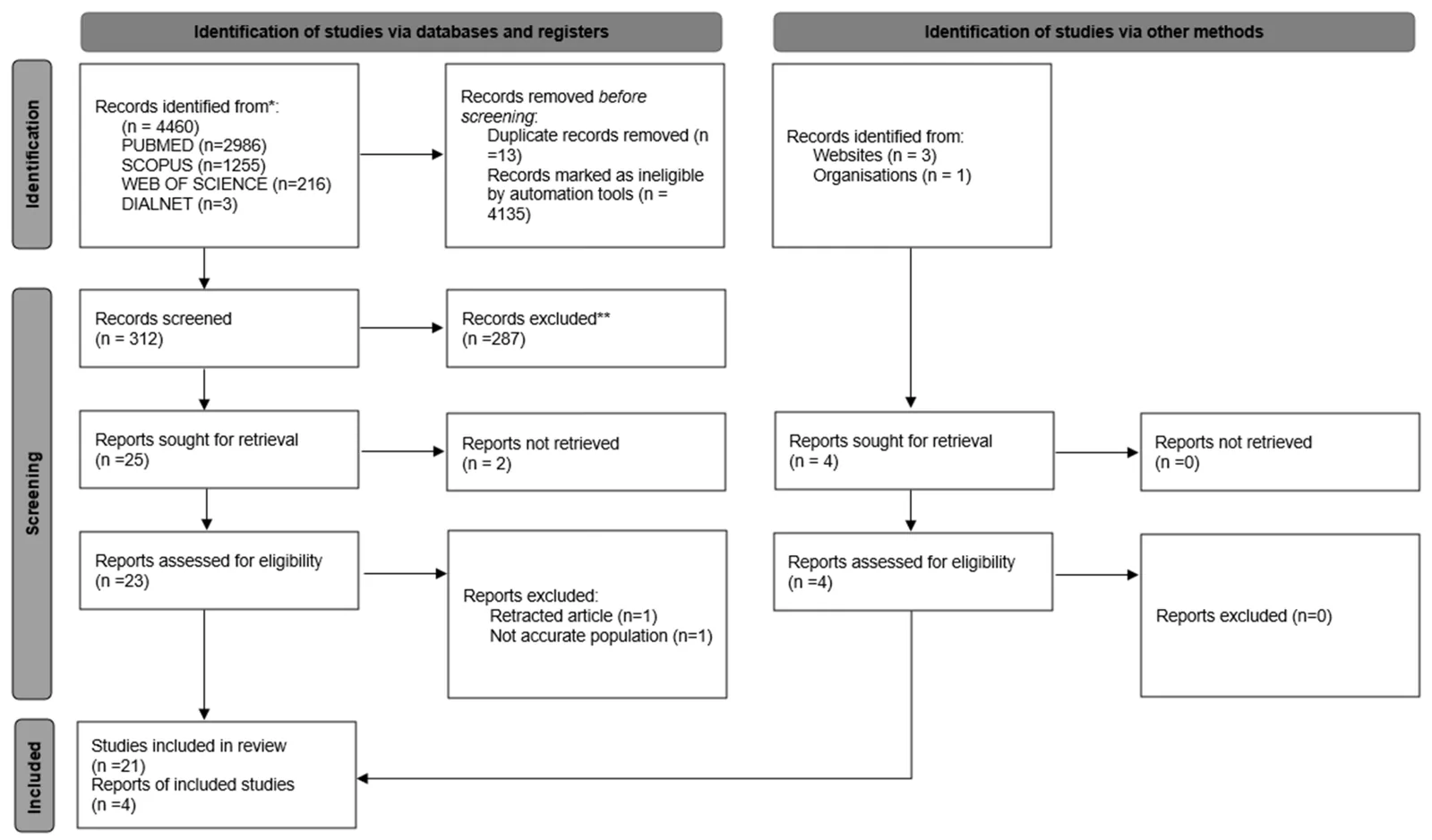
PRISMA flow diagram. Note: * and ** indicate the total.

Data extraction was independently performed by the same reviewers using a standardized data extraction form developed for this review. Extracted information included study characteristics, participant demographics, study design, intervention type, pain assessment instruments, individualized therapeutic strategies, and the main findings relevant to the review objectives. The use of independent reviewers and a standardized extraction form was intended to reduce the risk of selection and extraction bias while enhancing the consistency and transparency of the review process (Figure 1).

### 2.4. Data Synthesis

Given the methodological heterogeneity of the included studies, a narrative thematic synthesis was undertaken. Studies were grouped according to predefined themes aligned with the objectives of the review, including pain perception and expression, pain assessment tools, pharmacological interventions, non-pharmacological interventions, individualized therapeutic environments, and implications for nursing practice. Patterns, consistencies, and differences across studies were identified and narratively synthesized. This approach enabled comparison of findings across different study designs while preserving the contextual complexity of the available evidence.

### 2.5. Methodological Quality Appraisal

In accordance with the objectives and methodological framework of a scoping review and the PRISMA-ScR recommendations, a formal critical appraisal of the methodological quality or risk of bias of the included studies was not performed, as the purpose of this review was to map and synthesize the nature and extent of the available evidence rather than determine the effectiveness of interventions.

### 2.6. Additional Sources

To complement the database search, relevant clinical guidelines and authoritative documents were consulted, including those from the International Association for the Study of Pain (IASP), the Spanish Society of Pediatric Emergencies (SEUP), and the Diagnostic and Statistical Manual of Mental Disorders, Fifth Edition (DSM-5). These sources were used for contextual purposes and were not included as primary studies in the evidence synthesis.

### 2.7. Protocol and Reporting

The review protocol was not prospectively registered in a publicly accessible database. Although prospective registration is encouraged to enhance transparency and reduce the risk of reporting bias, it was not undertaken for this review. Nevertheless, the review was conducted according to a predefined methodology established prior to study selection and data extraction and was reported in accordance with the PRISMA Extension for Scoping Reviews (PRISMA-ScR) guidelines.

## 3. Results

The included studies addressed pain assessment and management in children with ASD across a range of clinical settings and methodological designs. Most studies were observational and focused on procedural pain, whereas interventional studies were less frequent. Despite methodological heterogeneity, the evidence consistently highlighted the need for individualized approaches to pain assessment and management. The findings were synthesized into three thematic domains aligned with the objectives of this review: pain assessment strategies, pain management interventions, and individualized therapeutic environments supporting pain management and coping.

### 3.1. Pain Assessment Strategies

Pain assessment emerged as one of the most extensively investigated topics across the included studies. The evidence consistently indicates that no single assessment method is sufficient to accurately identify pain in children with ASD because of the marked heterogeneity in communication abilities, sensory processing, and behavioral responses. Consequently, current evidence supports a multimodal assessment approach that integrates self-report measures, observational tools, caregiver input, and physiological indicators, with the selection of assessment strategies tailored to each child’s individual characteristics, communication abilities, and clinical context.

#### 3.1.1. Self-Report Measures

Self-report remains the preferred method when cognitive and communicative abilities allow it. Common tools include: Numerical Rating Scales, Verbal Descriptor Scales, Visual Analog Scales (VAS) and Facial expression scales (e.g., Wong-Baker Faces, FPS-R, Oucher). However, evidence indicates limitations in ASD populations due to difficulties in abstract reasoning, symbolic interpretation, and emotional labeling, which may reduce the reliability of these tools [9,15].

#### 3.1.2. Observational and Behavioral Scales

When self-report is not feasible, behavioral scales are widely used. The most frequently reported instruments include: FLACC-revised scale, Non-Communicating Children’s Pain Checklist-Revised (NCCPC-R), Pediatric Pain Profile (PPP) and Individualized Numeric Rating Scale (INRS). These tools assess pain through behavioral and physiological indicators such as facial expression, motor activity, vocalization, and consolability. However, most were not specifically designed for ASD populations, limiting their sensitivity to atypical pain expression patterns [20,21].

#### 3.1.3. Caregiver-Based Assessment

Caregiver input is consistently identified as a critical component of pain assessment in ASD. Parents and caregivers provide essential information regarding baseline behavior, enabling identification of deviations suggestive of pain. The Individualized Numeric Rating Scale (INRS) is one example of a tool incorporating caregiver-defined behavioral anchors. Despite its value, caregiver-based assessment introduces subjectivity and variability depending on caregiver experience and interpretation [20,22].

#### 3.1.4. Physiological Indicators

Physiological parameters such as heart rate, blood pressure, and skin conductance are reported as complementary measures. However, their specificity for pain is limited, as they may also reflect anxiety, sensory overload, or environmental stressors, which are highly prevalent in ASD populations [20,23].

Overall, the literature supports a multidimensional approach to pain assessment in children with ASD. Although several instruments are available, none has demonstrated sufficient psychometric validation for exclusive use in this population. Individualized assessment combining multiple information sources therefore remains the most consistently recommended strategy.

### 3.2. Pain Management Interventions

Pain management in children with ASD is consistently described within a biopsychosocial framework integrating pharmacological and non-pharmacological interventions. Across the included studies, individualized management strategies tailored to each child’s developmental level, communication abilities, sensory profile, and clinical needs were associated with improved pain management and reduced procedural distress. The evidence also emphasizes the importance of combining these interventions with family involvement and multidisciplinary care to optimize clinical outcomes. However, the available evidence remains methodologically heterogeneous and is predominantly derived from observational studies, limiting the strength of current recommendations.

#### 3.2.1. Pharmacological Interventions

Pharmacological management follows standard pediatric analgesic principles, with treatment selection based on pain intensity and clinical context. Commonly reported medication classes include non-opioid analgesics and opioids for moderate to severe pain. The limited literature highlights variability in dosing considerations due to age, weight, comorbidities, and potential off-label use in pediatric settings, underscoring the heterogeneity of pharmacokinetic and pharmacodynamic responses in children [24].

#### 3.2.2. Non-Pharmacological Interventions

Non-pharmacological interventions can be categorized into three main groups: behavioral, physical, and cognitive strategies. Across all categories, active participation of the child and the systematic use of positive reinforcement are considered essential components of effective implementation.

##### Behavioral Interventions

Behavioral strategies aim to support children in developing skills to manage pain and anxiety. The literature describes the following techniques:

Deep breathing exercises: These techniques reduce perceived pain intensity through activation of the parasympathetic nervous system. In younger children, they are commonly introduced using simple metaphors (e.g., “blowing up an imaginary balloon”), whereas older children benefit from structured breathing patterns, such as inhaling for 4 s and exhaling for 4 s. Evidence suggests potential benefits when these techniques are implemented within structured pre-procedural routines; however, ASD-specific evidence remains limited [3].

Relaxation techniques: These include progressive muscle relaxation and guided imagery. In children with ASD, the literature emphasizes the importance of concrete instructions and prior rehearsal to improve comprehension and intervention efficacy [18].

Coping skills training: This intervention focuses on developing individualized coping plans for procedural or painful experiences. It is typically designed collaboratively by multidisciplinary teams, children, and caregivers prior to hospital procedures. Its implementation is based on positive reinforcement and early identification of anxiety responses to enable timely redirection [3].

##### Physical Interventions

Physical strategies described in the literature for pediatric pain management include:

Transcutaneous Electrical Nerve Stimulation (TENS) involves electrode placement over or near the painful area, delivering high-frequency, low-intensity electrical stimulation for a limited period (typically up to 20 min). Evidence from pediatric procedural pain studies suggests that TENS can reduce pain perception through modulation of peripheral and central nociceptive pathways, with some studies reporting early onset of analgesic effects during application. However, in children with ASD, direct evidence remains limited, and its use is generally extrapolated from broader pediatric populations. Its applicability in ASD may be influenced by individual sensory processing differences and tolerance to electrical or tactile stimulation, highlighting the need for individualized assessment and monitoring [20].

Cryotherapy may be used as an adjunct non-pharmacological intervention in pediatric procedural pain. However, in children with ASD, its application should be individualized due to altered sensory processing and variable responses to thermal stimuli. While evidence supports its analgesic effect in general pediatric populations, ASD-specific data remain limited and largely extrapolated from neurotypical samples [3].

##### Cognitive Interventions

Cognitive strategies aim to redirect attention away from painful stimuli, thereby reducing perceived pain and anxiety. The most commonly reported interventions include distraction techniques.

Distraction techniques include a range of cognitive and sensory-based interventions such as music, videos, toys, and interactive digital tools, selected according to the child’s developmental stage, cognitive functioning, and individual sensory profile. In children with ASD, current evidence emphasizes the importance of sensory-informed and individualized approaches, as sensory processing differences significantly influence attention, engagement, and tolerance to external stimuli. Caregiver input is particularly relevant in this population to identify sensory preferences, reduce overstimulation, and tailor interventions during medical or procedural care [7,25,26].

Among these approaches, virtual reality (VR) has emerged as a promising non-pharmacological intervention for the management of procedural pain and anxiety in pediatric populations. VR provides immersive, multisensory environments that facilitate attentional redirection during painful procedures, thereby reducing the cognitive and emotional processing of nociceptive stimuli. Systematic reviews in pediatric populations indicate reductions in pain, anxiety, and procedural distress; however, the applicability of these findings to children with ASD requires further investigation [27,28].

However, the implementation of distraction-based interventions, including VR, requires careful individualization. Variability in sensory processing, potential susceptibility to sensory overload, and differences in tolerance to immersive audiovisual stimuli may influence feasibility and effectiveness. Despite these considerations, emerging evidence suggests that VR may be well accepted when appropriately adapted, particularly when predictable, structured, and sensory-modulated environments are used. Nevertheless, ASD-specific empirical evidence remains limited, and most conclusions are extrapolated from general pediatric populations [29,30].

Examples of VR-based interventions identified in the literature include Golden Breath [16], Dr. Zoo [31], and Hospital Adventure [30]. These interventions integrate gamification and immersive technologies to reduce procedural anxiety and pain perception in pediatric populations, with additional reported benefits on caregiver anxiety and patient knowledge acquisition. Despite these reported benefits, the literature highlights the need for further studies to establish the effectiveness of VR-based interventions and to standardize their implementation. Training of healthcare professionals, particularly pediatric nurses, is consistently identified as a key factor for optimizing clinical use [30].

Animal-assisted interventions have been associated with potential benefits in reducing perceived pain and distress in pediatric populations, particularly during medical procedures. However, evidence regarding their effects on anxiety remains heterogeneous, with variability across study designs, intervention protocols, and outcome measures. Overall, current findings should be interpreted with caution due to methodological limitations, small sample sizes, and lack of standardization. Consequently, further high-quality randomized controlled trials are needed to establish their efficacy and clinical applicability in pediatric pain management [32].

Overall, current evidence supports combining pharmacological and non-pharmacological interventions within individualized management plans. Nevertheless, the effectiveness of many interventions remains supported by limited evidence, highlighting the need for well-designed pediatric studies focused specifically on children with ASD.

### 3.3. Individualized Therapeutic Environments

Individualized therapeutic environments emerged as a key cross-cutting component influencing both pain assessment and pain management in children with ASD. Across the included studies, environmental adaptations addressing sensory processing differences, communication needs, predictability, and caregiver involvement were consistently associated with reduced distress, improved cooperation during healthcare encounters, and more individualized care. Sensory-adapted environments may also facilitate more accurate pain recognition by minimizing behavioral responses related to anxiety and sensory overload, although this potential benefit has not yet been confirmed by robust comparative studies.

Healthcare environments are frequently perceived as distressing by children with autism due to sensory hypersensitivity, unpredictability, and disruption of established routines [15]. Consequently, individualized therapeutic environments aim to reduce sensory load and support emotional regulation through tailored, patient-centered environmental modifications. These adaptations commonly include a reduction in auditory and visual stimuli (e.g., control of noise and lighting), provision of familiar objects and comfort items, maintenance of structured and predictable routines, use of visual supports and pictographic communication systems, adaptation of clinical and waiting areas to sensory needs, and active involvement of caregivers throughout care delivery [33,34].

The Autistic SPACE framework highlights sensory needs, predictability, acceptance, communication, and empathy as core principles of autism-informed healthcare delivery. Emerging evidence suggests that sensory-adapted and structured environments may reduce anxiety, distress, and behavioral dysregulation in autistic children, while also improving cooperation during medical procedures. However, the current evidence base remains limited and heterogeneous, with a predominance of observational and feasibility studies, which limits the strength of conclusions and their generalizability [33,34].

Importantly, individualized environmental adjustments may also enhance the accuracy of pain assessment by reducing confounding factors such as sensory overload, fear, and stress-related behavioral responses, which may otherwise be misinterpreted as pain expression. Given that sensory processing differences significantly influence behavioral reactivity in clinical contexts, distinguishing pain from environmental distress remains challenging. Consequently, sensory-adapted and structured healthcare environments may support more reliable interpretation of behavioral cues by minimizing non-nociceptive triggers [26,34]. However, although this potential benefit is theoretically plausible, it has not yet been demonstrated through robust comparative studies, and further research is required to determine its specific impact on pain assessment accuracy in this population.

In summary, the findings suggest that individualized therapeutic environments represent an important complement to conventional pain assessment and management strategies in children with ASD by reducing sensory overload, procedural distress, and barriers to effective communication during healthcare encounters. Although current evidence supports their potential clinical benefits, it is largely based on observational and feasibility studies. Therefore, robust comparative studies are needed to determine the effectiveness of specific environmental adaptations, identify the most beneficial strategies across different healthcare settings and patient profiles, and support their standardized implementation in pediatric practice.

## 4. Conclusions

This scoping review provides a comprehensive synthesis of the available evidence on pain assessment and management in children with ASD, highlighting the complexity of pain recognition, the importance of individualized therapeutic environments, and the need for multidisciplinary approaches to optimize care. Although research interest has increased in recent years, the available literature remains fragmented, methodologically heterogeneous, and predominantly based on observational studies, small sample sizes, and diverse outcome measures. Consequently, the overall strength of the evidence is low to moderate, limiting the certainty of evidence underpinning current clinical recommendations.

In addition, as this review followed the methodological framework of a scoping review, no formal methodological quality appraisal or risk-of-bias assessment was performed. Accordingly, the findings should be interpreted as a comprehensive mapping of the available evidence rather than as an assessment of the certainty or effectiveness of individual interventions.

The absence of pain assessment instruments specifically developed and psychometrically validated for children with ASD remains one of the most clinically relevant findings. Current practice continues to rely on tools developed for neurotypical children or broader neurodevelopmental populations, which may contribute to inaccurate pain assessment and under recognition of pain. Furthermore, the lack of standardized, ASD-specific clinical protocols adapted to the sensory, communicative, and behavioral characteristics of this population contributes to considerable variability in clinical practice.

Although individualized therapeutic environments and non-pharmacological interventions, particularly sensory-based and behavioral strategies, are consistently associated with improved pain management, the available evidence is heterogeneous and largely observational. Therefore, these approaches should be considered promising components of individualized care rather than interventions supported by high-quality evidence. Similarly, emerging technologies such as virtual reality show encouraging preliminary results for reducing procedural pain and anxiety, but further well-designed pediatric studies are needed to establish their effectiveness and long-term clinical value.

Overall, the findings support an individualized, multidisciplinary approach to pain management while underscoring the need for high-quality research to strengthen the evidence base. Future studies should prioritize the development and psychometric validation of ASD-specific pain assessment instruments, the evaluation of standardized clinical protocols, and adequately powered comparative studies assessing pharmacological, non-pharmacological, and technology-assisted interventions. Within this context, nurses play a pivotal role in systematic pain assessment, individualized care planning, family-centered support, and multidisciplinary coordination. Nevertheless, further evidence is required to establish which nursing interventions provide the greatest clinical benefit and should be incorporated into standardized models of care.

## Data Availability

The data presented in this study are available on request from the corresponding author. The data are not publicly available due to privacy and ethical reasons.

## Supplementary Materials

The following supporting information can be downloaded at: https://www.mdpi.com/article/10.3390/children13080979/s1, Supplementary Material S1: Search Strategy.
