# Supplementary material for: Individualized Therapeutic Environments for Pain Management in Children with Autism Spectrum Disorder: A Scoping Review
Source: Children (Basel). 2026 Jul 23;13(8):979. doi: 10.3390/children13080979 (PMC13510502; doi:10.3390/children13080979)
Supplement: Supplementary file 1 [file children-13-00979-s001.zip › children-4401904-supplementary.pdf]

Supplementary Materials S1. Search Strategy

Complete Database-Specific Search Strategies

The literature search was conducted in accordance with the PRISMA-ScR recommendations. Electronic searches were performed in PubMed, Scopus, and Web of Science. Searches were restricted to studies published between January 2020 and December 2025 and to articles published in English or Spanish. No restrictions were applied regarding study design. The search strategy combined controlled vocabulary (MeSH, where applicable) and free-text terms related to autism spectrum disorder, pain assessment and management, pediatric populations, nursing, caregivers, and healthcare. Database-specific syntax and Boolean operators were adapted for each database.

1. Information Sources

- PubMed
- Scopus
- Web of Science Core Collection

2. Search Concepts

Concept	Search terms
Population	Autism Spectrum Disorder; ASD
Pain	Pain Management; Pain Perception
Pediatric population	Pediatrics; Child
Healthcare	Nursing Research; Pediatric Nursing; Health Care
Caregivers	Caregiver

3. PubMed

The PubMed strategy combined MeSH terms and free-text terms.

- Search 1. MeSH-based strategy

((((("autism spectrum disorder"[MeSH Terms]) AND "Pain management"[MeSH Terms]) OR "pain perception"[MeSH Terms]) AND "pediatrics"[MeSH Terms]) OR "child"[MeSH Terms]) AND "Nursing Research"[MeSH Terms])

- Search 2. Free-text strategy

((((("autism spectrum disorder"[All Fields] OR "ASD"[All Fields]) AND ("Pain management"[All Fields] OR "pain perception"[All Fields]) AND "pediatric*"[All Fields]) OR "child*"[All Fields]) AND "nurs*"[All Fields]))

- Search 3. Combined strategy

((((("autism spectrum disorder"[MeSH Terms] AND "Pain management"[MeSH Terms]) OR "pain perception"[MeSH Terms]) AND "pediatrics"[MeSH Terms]) OR "child"[MeSH Terms]) AND "Nursing Research"[MeSH Terms] AND (((("autism spectrum disorder"[All Fields] OR "ASD"[All Fields]) AND ("Pain management"[All Fields] OR "pain perception"[All Fields]) AND "pediatric*"[All Fields]) OR "child*"[All Fields]) AND "nurs*"[All Fields]))

Applied limits:

• Publication date: January 2020–December 2025

• Language: English or Spanish

4. Scopus

The search strategy was adapted to the Scopus syntax.

(ALL("autism spectrum disorder") OR ALL("ASD") AND ALL("Pain management") OR ALL("pain perception") AND ALL("pediatric*") OR ALL("child*") AND ALL("nurs*"))

Applied limits:

• Publication years: 2020–2025

• Language: English or Spanish

5. Web of Science

The search strategy was adapted to the Web of Science syntax.

TS=((autism OR "autism spectrum disorder" OR ASD) AND ("pain management" OR "pain perception" OR pain) AND (pediatric* OR child* OR adolescen*) AND (nurs* OR "nursing research" OR "health care" OR caregiv*))

Applied limits:

• Publication years: 2020–2025

• Language: English or Spanish

6. Search Management

Duplicate records were removed before screening. Study selection was performed independently by two reviewers according to predefined eligibility criteria. Any disagreements were resolved through discussion until consensus was reached.
